# Encysted Hydrocele of Spermatic Cord in Adult: A Rare Case Report

**DOI:** 10.7759/cureus.47782

**Published:** 2023-10-27

**Authors:** Yashraj Jain, Ranjana Lanjewar, Simran Chauhan

**Affiliations:** 1 General Surgery, Jawaharlal Nehru Medical College, Wardha, IND

**Keywords:** encysted hydrocele, painless scrotal mass, benign scrotal mass, scrotal surgery, testicular mass, scrotal swelling, primary hydrocele

## Abstract

Hydrocele represents a commonly encountered pathological condition characterized by the development of scrotal swelling. In contrast, spermatic cord hydrocele is a relatively rare condition that arises from the abnormal closure of the processus vaginalis. This condition entails the accumulation of fluid within the spermatic cord, and although hydrocele itself is a frequently observed ailment affecting roughly 100 out of 100,000 men, encysted hydrocele of the spermatic cord is a seldom-seen variant. Furthermore, it is noteworthy that spermatic cord hydrocele tends to be more prevalent in the pediatric population compared to adults. In our clinical practice, a patient presented with swelling localized to the right hemi-scrotum and was initially diagnosed as suffering from a chronic right-sided hydrocele of the tunica vaginalis. However, during the surgical intervention, the true nature of the condition became evident, as it was determined to be an encysted hydrocele of the cord. This case report chronicles the diagnostic journey surrounding hydrocele, highlighting the distinction in surgical management between encysted hydrocele and vaginal hydrocele of the testis.

## Introduction

A hydrocele is characterized by an accumulation of excess fluid between the two layers of the tunica vaginalis, namely, the parietal layer and the visceral layer. Spermatic cord hydrocele, on the other hand, is a condition resulting from the incomplete closure of the processus vaginalis [1]. Notably, approximately 10% of newborns are affected by hydroceles, which tend to resolve within the first year of life [2]. Hydrocele of the spermatic cord is an unusual anomaly pertaining to the spermatic cord, stemming from the inadequate closure of the processus vaginalis [3]. This condition can be classified into two types: communicating hydrocele, which is in communication with the peritoneal cavity, and encysted spermatic hydrocele, which lacks such communication [2].

A comprehensive five-year prospective study conducted at the Department of Surgery, University of Benin Teaching Hospital in Benin City, Nigeria, revealed that out of 163 children, communicating hydrocele accounted for 75 cases (46.0%), while non-communicating hydrocele constituted 88 cases (54.0%) [4]. The differentiation of various hydrocele types is imperative for appropriate management and favorable perioperative outcomes. The presence of fluid brilliantly transilluminates the swelling, aiding in the differentiation from conditions such as an inguinal hernia, spermatic cord lipoma, epididymal cyst, spermatocele, testicular tumor, scrotal edema, and varicocele [5].

Diagnosis of hydrocele primarily relies on clinical examination, encompassing scrotal swelling, the absence of a cough impulse, and the ability to manipulate the swelling, which distinguishes it from a hernia. Ultrasound sonography (USG) can serve as a confirmatory diagnostic tool, enabling the assessment of testicular echotexture. On ultrasound, hydrocele typically presents as an anechoic area enveloping the testis [6]. The type and size of hydroceles can also be characterized through ultrasound examination [7]. It is important to perform ultrasound examinations in both the standing and supine positions, as communicating hydroceles may reduce when the patient is lying down [7].

The primary mode of treatment for hydrocele is surgical intervention, typically reserved for large, symptomatic, or complicated hydroceles [7]. In cases of congenital hydrocele, herniotomy is considered if spontaneous reduction does not occur [7]. The choice of surgical technique may vary depending on the specific characteristics of the hydrocele; for instance, Lord's plication is commonly favored for thin-walled hydroceles, while Jaboulay's eversion of the sac is preferred for cases involving large hydroceles with thick sacs [7]. This case report offers a detailed account of the clinical examination and treatment of a 48-year-old adult who presented with a rare occurrence of an encysted spermatic cord hydrocele.

## Case presentation

A 48-year-old male presented to the hospital with a five-year history of swelling on the right side of his scrotum. The swelling had developed gradually without any sudden onset, and it had been progressively increasing in size. Notably, the swelling did not change in size during strenuous physical activities. The patient had no history of constipation, heavy weight lifting, chronic cough, increased frequency of urination, or nocturia. Upon thorough general and systemic examination, no significant abnormalities were observed. During abdominal examination, normal findings were noted, including regular bowel sounds and the absence of any significant abnormalities. A firm swelling measuring 5 x 4 x 2 cm was detected on the right scrotum on local examination. No cough impulse was elicited, and the right-sided spermatic cord was palpable. The swelling exhibited remarkable transillumination. Bilateral testes were palpable, and the skin over the swelling appeared tense without signs of local inflammation.

**Figure 1:**
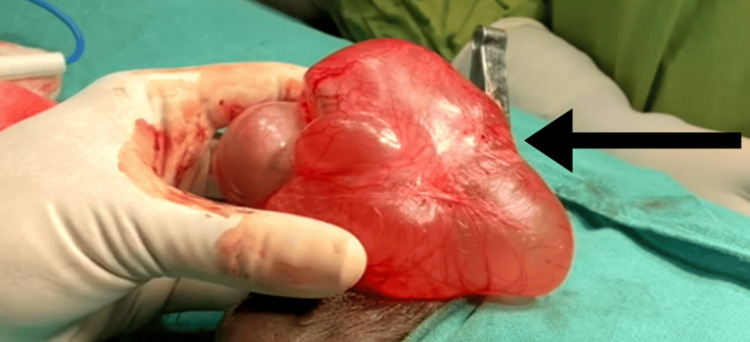
Sac and testis delivered out

A preliminary diagnosis of chronic right-sided vaginal hydrocele was made based on clinical examination. An ultrasound of the inguinoscrotal region supported this diagnosis. Laboratory findings were all within normal limits, and the patient was scheduled for a procedure to perform a right-sided Jaboulay eversion of the sac. Under spinal anesthesia, the patient was positioned supine. On the right side, a vertical incision was made, 2 cm lateral to the median raphe. The incision was extended to the sac wall, and the sac was carefully separated from the dartos muscle. The sac was then completely delivered (Figure 1).

**Figure 2:**
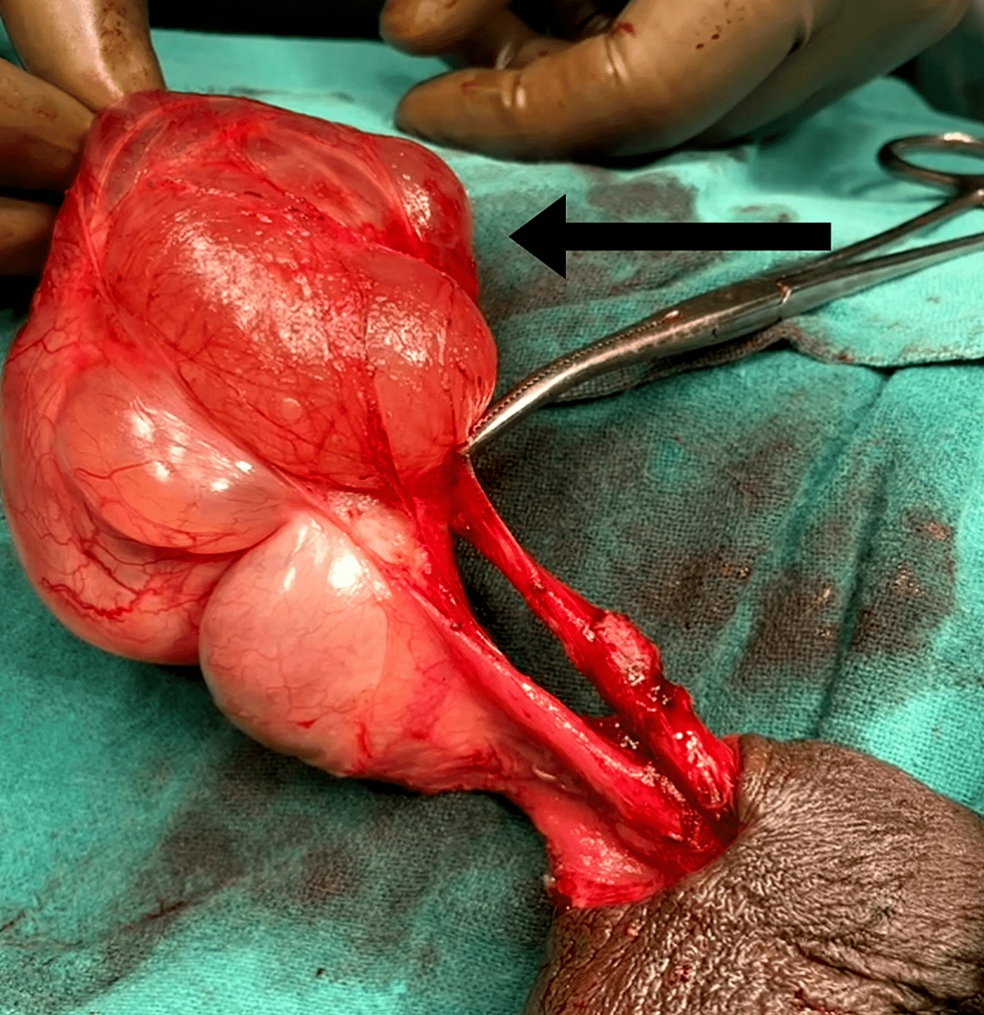
Sac excised from surrounding structures

**Figure 3:**
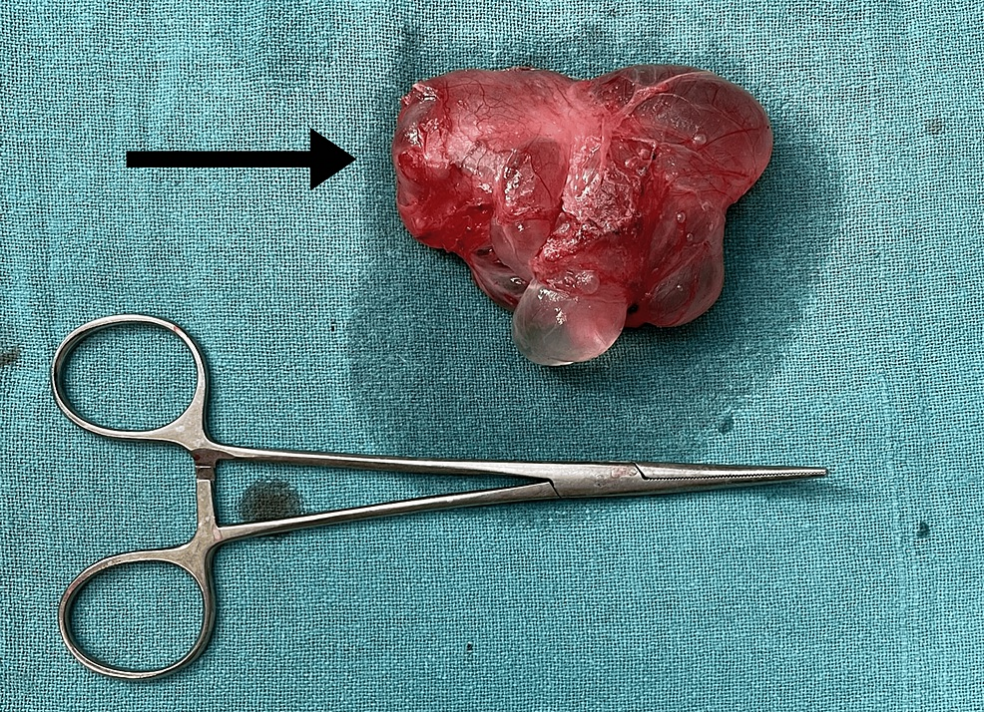
Excised specimen of encysted hydrocele of the cord

The patient's postoperative course was uneventful and deemed excellent. Intravenous antibiotics and analgesics were administered for two days, after which the patient was discharged with oral antibiotics and analgesics. Suture removal was performed 10 days after the surgery, and no wound complications were observed. A specimen was sent for histopathological examination, which revealed a loose connective tissue lined by flattened mesothelial cells with chronic inflammatory infiltrates, indicative of chronic hydrocele. Unfortunately, no histological slides are available for reference. Notably, a significant cystic swelling originating from the spermatic cord was observed. The testis was distinctly visible, and adhesions between the testis and hydrocele were successfully separated. The swelling was entirely excised (Figure 2 and Figure 3).

Hemostasis was effectively ensured, the testes were repositioned to their anatomical location, and the surgical site was closed in multiple layers. The excised specimen was sent for histopathological examination, supporting the chronic hydrocele diagnosis. The patient experienced a remarkable and uneventful recovery in the postoperative period.

## Discussion

A hydrocele is characterized by the accumulation of fluid between the parietal and visceral layers of the tunica vaginalis, which is a peritoneal cavity remnant situated in the scrotum [8]. During the development of a male fetus, the testis descends from the abdominal cavity to the scrotum at approximately 28-32 weeks of gestation. As it descends, it carries folds of the peritoneum known as the processus vaginalis. A spermatic cord hydrocele arises from the failure of proper closure at the two ends of the processus vaginalis [8]. Over time, the processus vaginalis undergoes involution, leaving only the distal segment, which ultimately forms the tunica vaginalis. A communicating hydrocele is formed when closure does not occur at both the proximal and distal ends [8]. Conversely, the formation of a funicular hydrocele arises when only the distal end remains open. Encysted hydrocele occurs when both ends are closed, and the segment in between fails to undergo involution.

Notably, most documented cases have predominantly involved pediatric patients, with relatively few publications focusing on adult cases [9]. Spermatic cord hydroceles can be challenging to differentiate clinically from other pathologies in the inguinal region. However, using ultrasound plays a crucial role in accurately distinguishing them from other diseases. Diagnosis typically relies on clinical evaluation and can be significantly aided by ultrasound, which boasts excellent sensitivity and the ability to differentiate hydroceles from other pathologies [10]. Ultrasound is especially effective in determining the location, size, and shape of spermatic cord hydroceles. Managing encysted hydroceles often involves surgical excision, which can be performed under local anesthesia [11].

## Conclusions

Encysted hydrocele of the cord in adults is indeed a rare condition, which, in some instances, may closely resemble an irreducible hernia or, in others, a chronic vaginal hydrocele. Despite its relative rarity, the significance of timely and proper management cannot be overstated, given that hydrocele, like any medical condition, necessitates prompt intervention to prevent potential complications. In the realm of surgical procedures, addressing hydrocele typically entails minor surgery, which is best executed promptly to mitigate risks. However, the situation takes an intriguing turn when dealing with encysted spermatic cord hydrocele, as the conventional methods of eversion or plication of the parietal layer of the tunica vaginalis prove ineffective in these cases. Instead, a more intricate approach becomes necessary, one involving thorough dissection and complete excision of the cystic swelling to ensure appropriate management. The rationale behind presenting this case report is to contribute to the still relatively sparse body of published cases and deepen our collective understanding of this unique medical condition. By delving into the specific characteristics, clinical outcomes, and any nuances that distinguish our case from others previously documented, we aim to shed light on the complexities and subtleties of encysted spermatic cord hydrocele in adults.
